# Distribution of Gene Mutations Associated with Familial Normosmic Idiopathic Hypogonadotropic Hypogonadism

**DOI:** 10.4274/Jcrpe.725

**Published:** 2012-09-11

**Authors:** Fatih Gürbüz, L. Damla Kotan, Eda Mengen, Zeynep Şıklar, Merih Berberoğlu, Sebila Dökmetaş, Mehmet Fatih Kılıçlı, Ayla Güven, Birgül Kirel, Nurçin Saka, Şükran Poyrazoğlu, Yaşar Cesur, Murat Doğan, Samim Özen, Mehmet Nuri Özbek, Hüseyin Demirbilek, M. Burcu Kekil, Fatih Temiz, Neslihan Önenli Mungan, Bilgin Yüksel, Ali Kemal Topaloğlu

**Affiliations:** 1 Çukurova University Faculty of Medicine, Department of Pediatric Endocrinology, Adana, Turkey; 2 Çukurova University Institute of Sciences, Department of Biotechnology, Adana, Turkey; 3 Ankara University Faculty of Medicine, Department of Pediatric Endocrinology, Ankara, Turkey; 4 Cumhuriyet University Faculty of Medicine, Department of Endocrinology, Sivas, Turkey; 5 Göztepe Educational and Research Hospital, Department of Pediatric Endocrinology, İstanbul, Turkey; 6 Osmangazi University Faculty of Medicine, Department of Pediatric Endocrinology, Eskişehir, Turkey; 7 İstanbul University Faculty of Medicine, Department of Pediatric Endocrinology, İstanbul, Turkey; 8 Yüzüncü Yıl University Faculty of Medicine, Department of Pediatric Endocrinology, Van, Turkey; 9 Mersin Children’s Hospital, Department of Pediatric Endocrinology, Mersin, Turkey; 10 Diyarbakır Children’s Hospital, Department of Pediatric Endocrinology, Diyarbakır, Turkey

**Keywords:** Normosmic idiopathic hypogonadotropic hypogonadism, gene, mutation

## Abstract

**Objective:** Normosmic idiopathic hypogonadotropic hypogonadism (nIHH) is characterized by failure of initiation or maintenance of puberty due to insufficient gonadotropin release, which is not associated with anosmia/hyposmia. The objective of this study was to determine the distribution of causative mutations in a hereditary form of nIHH.

**Methods:** In this prospective collaborative study, 22 families with more than one affected individual (i.e. multiplex families) with nIHH were recruited and screened for genes known or suspected to be strong candidates for nIHH.

**Results:** Mutations were identified in five genes (GNRHR, TACR3, TAC3, KISS1R, and KISS1) in 77% of families with autosomal recessively inherited nIHH. GNRHR and TACR3 mutations were the most common two causative mutations occurring with about equal frequency.

**Conclusions:** Mutations in these five genes account for about three quarters of the causative mutations in nIHH families with more than one affected individual. This frequency is significantly greater than the previously reported rates in all inclusive (familial plus sporadic) cohorts. GNRHR and TACR3 should be the first two genes to be screened for diagnostic purposes. Identification of causative mutations in the remaining families will shed light on the regulation of puberty.

**Conflict of interest:**None declared.

## INTRODUCTION

Idiopathic hypogonadotropic hypogonadism (IHH) is characterized by failure of initiation or maintenance of puberty due to insufficient gonadotropin release for unknown reasons. These patients do not develop secondary sexual characteristics and their reproductive system remains immature. When the embryonic migration of gonadotropin-releasing hormone (GnRH) neurons from the nasal placode to their final destination in the hypothalamus is disrupted, the resulting phenotype is Kallmann syndrome, which is clinically characterized by hypogonadotropic hypogonadism and anosmia, while the term normosmic IHH (nIHH) denotes those IHH cases which not associated with anosmia/hyposmia (1). nIHH-causing mutant genes in familial cases of nIHH have brought invaluable insight to the governance of the hypothalamo–pituitary–gonadal axis including puberty. Identification of mutations in consecutive patients in familial cases of nIHH in a set time period in a highly consanguineous society may provide clues for the specific genetic etiology as well as for the potential for novel genes taking part in the pubertal process. The prevalence of IHH has been estimated to be around 1-10/100 000 and known genetic defects have been reported to account for only 30-50% of all cases with nIHH (2,3). 

In this paper, we report all identified causative mutations in multiplex families with nIHH over the past seven years in Turkey. Our findings indicate that a significant proportion of familial cases are accounted for by five genes (GNRHR, TACR3, TAC3, KISS1R, and KISS1). 

## METHODS

The diagnosis of nIHH in this cohort was based on the absence of any signs of spontaneous puberty by age 13 in girls (Tanner breast stage 1) and by age 14 in boys (testicular volume <4 mL), a bone age of 11.5 years or greater, with concentrations of testosterone and estradiol at hypogonadal levels [<20 ng/dL (714 pmol/L) and <1.9 ng/dL (73 pmol/L), respectively] in the setting of inappropriately normal or low gonadotropin levels. All patients had normal levels of free thyroxine, thyroid stimulating hormone, prolactin, insulin-like growth factor-1, adrenocorticotropic hormone, and cortisol. They had no evidence of structural lesions on imaging of the hypothalamic-pituitary region. Cases with chronic systemic diseases (e.g. uremia, thalassemia, poorly controlled diabetes mellitus), eating disorders (e.g. anorexia nervosa, bulimia), or protein energy malnutrition were excluded from the study. Individuals with body mass indices corresponding to extreme thinness or obesity were also excluded. All subjects had a normal sense of smell on conventional testing. No case had features suggestive of Bardet-Biedl, Biemond, Prader-Willi, or any other syndromes. The cohort included consecutive patients who were recruited through the collaboration of several national institutions in Turkey, over the past seven years. The study was approved by the ethics committee of Çukurova University Faculty of Medicine. Written consent was obtained from all patients or their legal guardians, if under 18 years of age.

**Clinical and Hormonal Studies**

Serum luteinizing hormone (LH), follicle-stimulating hormone (FSH), estradiol, and testosterone levels were measured by immunofluorometric assays. A GnRH stimulation test (2.5 μg/kg, maximum 100 μg, i.v.) was performed in all subjects, and serum LH and FSH levels were measured at 0, 15, 30, 45, and 60 min after GnRH stimulation. Olfactory bulbs and sulci and hypothalamic-pituitary structures were analyzed by magnetic resonance imaging.

**DNA Sequencing**

DNA was extracted from blood leukocytes using standard methods. The coding regions and neighboring intronic regions of the known or strong candidate genes for nIHH (GNRHR, GNRH1, TACR3, TAC3, KISS1R, and KISS1) were amplified by polymerase chain reaction (PCR). The PCR products were purified and directly sequenced using Big Dye terminator cycle sequencing ready reaction kit (PE Applied Biosystems, Foster City, Calif., USA) in an ABI PRISM 310 automatic sequencer. 

Functional consequences of the novel mutations were predicted using the Polyphen-2 program (http://genetics.bwh.harvard.edu/pph2/). 

## RESULTS

**Figure 1:**
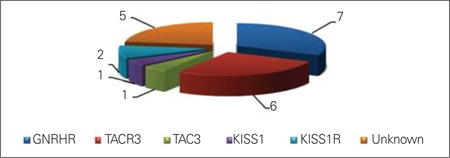
Distribution of the causative mutations in 22 families of patients with normosmic idiopathic hypogonadotropic hypogonadism

All patients presented with the signs and symptoms of a typical complete nIHH phenotype. We identified causative mutations in 17 out of 22 (77.2%) multiplex families with nIHH. The most common two causes were GNRHR and TACR3 mutations. Among the 22 families, we have identified mutations of GNRHR in seven (31.8%), TACR3 in six (27.2%), TAC3 in one (4.5%), KISS1 in one (4.5%), and KISS1R in two (9%). In all of these families, the mutations segregated within the respective nuclear families according to the rules of autosomal recessive inheritance. In five families, we have not been able to find mutations in any of these genes (Figure 1, Table 1). A summary of the findings in the 17 families is given below. 

**Family 1:** We have previously reported this family and its two members (two sisters) with absent breast development and primary amenorrhea due to p.R139C mutation in the GNRHR gene (4).

**Family 2:** The proband was a 15.4-year-old boy who presented with small testes. His two elder brothers were of the same clinical phenotype. Their parents were healthy cousins. The proband’s height was 177.5 cm and he weighed 59 kg. His pubic hair and axillary hair were at Tanner stage 3 and 2, respectively. Testis volume was 2 mL bilaterally. His stretched penile length was 4 cm. His bone age was 13 years. His basal testosterone level was <0.01 ng/dL. LH and FSH levels were 0.11 and 0.23 mIU/mL, respectively. A GnRH stimulation test produced maximal LH and FSH levels of 1.2 and 1.6 mIU/mL, respectively. The patient had a normal male karyotype. In this family also, the mutation analysis revealed p.R139C mutation in the GNRHR gene. 

**Family 3:** The proband was a 14.3-year-old boy who presented with micropenis and small testes. His past medical history was unremarkable except for left orchidopexy surgery at the age of one year. The parents were healthy distant cousins. The proband was 150 cm tall and weighed 53 kg. Both his pubic hair and axillary hair were at stage 2. His testes were bilaterally in the scrotum and measured 3 mL. His stretched penile length was 4 cm. His bone age was 13.6 years. Magnetic resonance imaging of the brain revealed normal olfactory bulbs and sulci and normal hypothalamic-pituitary structures. His basal testosterone level was <20 ng/dL with LH and FSH levels of 0.22 and 0.9 mIU/mL, respectively. A GnRH stimulation test produced a maximal LH level of 8.1 mIU/mL. He had a normal male karyotype. His 19-year-old sister was reported to have absent breast development and primary amenorrhea. The patients in family 3 showed p.N10K (from mother) and p.Q11K (from father) compound heterozygous mutations in the GNRHR gene.

**Family 4:** The proband was a 29-year-old man who had presented at age 16 with micropenis and small testes. His two elder brothers were of the same clinical phenotype. Their parents were not related. The proband’s height was 182 cm and he weighed 69 kg. His pubic and axillary hair was at Tanner stage 1. His testes were 3 mL bilaterally in the scrotum. His stretched penile length was 3 cm. His bone age was 18 years. His basal testosterone level was 40 ng/dL with LH and FSH levels of 1.31 and 1.4 mIU/mL, respectively. A GnRH stimulation test produced maximal LH and FSH levels of 8.1 and 4.89 mIU/mL, respectively. He had a normal male karyotype. The patients in family 4 showed p.R262Q (from mother) and p.X329W (from father) compound heterozygous mutations in the GNRHR gene.

**Family 5**: The proband was a 2-month-old boy who presented with micropenis of 1.6 cm and small testes. His past medical history was unremarkable. He was born after an uncomplicated pregnancy with a 3.2-kg birth weight. A 3-year-8-month-old brother was of the same clinical phenotype. Their parents were healthy cousins. This family has p.R139H mutation in the GNRHR gene.

**Family 6:** The proband was a 27-year-old man who had presented with small testes at age 15. His parents were healthy cousins. His pubic and axillary hair was at Tanner stage 3. His testes were 3 mL bilaterally in the scrotum. His stretched penile length was 6 cm. His testosterone level was 82 ng/dL with LH and FSH levels of 0.18 and 1.06 mIU/mL, respectively, while on treatment with monthly testosterone injections. A GnRH stimulation test produced maximal LH and FSH levels of 0.69 and 0.93 mIU/mL, respectively. He had a normal male karyotype. His 2 years older brother was reported to have a similar phenotype. The patients in this family have the novel mutation of p.M131T in the GNRHR gene. Polyphen 2 analysis indicated that the mutation p.M131T is probably damaging with a score of 0.99 out of 1.0.

**Family 7:** The proband was an 18.3-year-old boy who presented with micropenis and small testes. One younger brother was of the same clinical phenotype. Their parents were not related. The proband was 180 cm tall and weighed 51 kg. His pubic hair and axillary hair were at Tanner stage 3. His testes were 2 mL bilaterally in the scrotum. His stretched penile length was 3.7 cm. His bone age was 13 years. His basal testosterone level was 28.8 ng/dL with LH and FSH levels of <0.07 and 0.18 mIU/mL, respectively. A GnRH stimulation test produced maximal LH and FSH levels of 0.22 and 0.81 mIU/mL, respectively. He had a normal male karyotype. Family 7 has the novel mutation of p.L117R in the GNRHR gene. Polyphen 2 analysis indicated that the mutation p.L117R is probably damaging with a score of 0.97 out of 1.0.

**Families 8, 9, 10, and 11:** We have previously reported TACR3 mutations in these families (5, 6). 

**Families 12 and 13:** These two unrelated families have the same novel missense mutation in the TACR3 gene. Detailed reports of these two families have been submitted for publication (unpublished date).

**Family 14:** We have previously reported p.M90T mutation in the TAC3 gene in this family (5).

**Family 15:** We have previously reported p.N115K mutation in the KISS1 gene in this family (7). 

**Families 16 and 17:** These two unrelated families have the same novel missense mutation in the KISS1R gene. Detailed reports of these two families have been submitted for publication (unpublished date). 

## DISCUSSION

Known genetic defects have been reported to account for about 30-50% of all IHH cases (2,3) In large cohorts, the percentage of familial cases appear to vary between 6.3 to 25 (8,9,10). It is well known that the possibility of finding a gene mutation is significantly greater for familial cases as compared to sporadic ones. For example, KISS1R mutations were found as 3 out of 180 (1.6%) in sporadic cases, while this frequency was 5 out of 24 (20.8%) in familial cases (11). However, this distinction between sporadic and familial cases is not clear in all studies. Au et al (12) found mutations in 44% of a large cohort consisting of IHH patients. It is not clear how many of those probands came from multiplex families nor how many of those probands were cases of nIHH. In this study, we therefore focused exclusively on well described and almost uniform familial cases. Multiplex families with nIHH are more likely to be encountered in the Turkish population, which has a consanguinity rate of 21%, than in western societies (13). Consistently, in this study, we report identification of causative mutations in 17 families out of 22 families (77.2%) with nIHH, a finding which reflects a significantly higher percentage as compared to previous studies (2,3). Mutations in five genes (GNRHR, TACR3, TAC3, KISS1R, and KISS1) account for about three quarters of families with nIHH cases. Two genes, GNRHR and TACR3, are most likely to be found among familial cases. Therefore, these should be the first two genes to be screened in the molecular genetic diagnosis of nIHH.

Large scale screening indicates that GNRHR mutations account for 3.5-16% of sporadic cases of nIHH and up to 40% of familial cases (14). Beranova et al (15) reported GNRHR mutations in two of the 5 families with autosomal recessive pedigrees. In our study, we identified GNRHR gene mutations in seven of 22 families (31.8%) with nIHH. Thus, in our study, the rate of identified **GNRHR** gene mutations in nIHH phenotype was approximately the same as the previously reported rates for familial cases.

Most of the mutations (5 of 7) in GNRHR have been previously reported. To date, 22 mutations in GNRHR have been described; the most common mutations were Q106R and R262Q (15,16,17,18,19,20,21,22,23). The p.R139C mutation in the GNRHR gene was reported by one of us in family 1 (4), and family 2 harbored the same mutation (p.R139C). These two families are not known to be related to each other. The p.R139H mutation found in family 5 in the GNRHR gene has also been previously reported in Brazilian and Polish patients with nIHH (17, 24). As the patients in family 5 have a mutation in the same residue as family 1 and 2, arginine at position 139 could be considered a mutational hotspot. Chevrier et al (25) observed 17 of 269 patients bearing compound heterozygote or homozygote GNRHR mutations. However, in this report, it is not clear how many of those patients came from multiplex families. 

The p.R262Q in the GNRHR gene has previously been reported in homozygous or compound heterozygous state (16,18,19,22,26,27,28). In our family 4, R262Q was in compound heterozygous state with p.X329WextX22, which is a novel mutation predicted to be deleterious due to 22 amino acid longer derivative protein. Likewise, compound heterozygous states for p.N10K and p.Q11K mutations in GNRHR gene have been reported previously (29).The p.M131T mutation in family 6 and p.L117R mutation in family 7 in the GNRHR gene have not been reported previously. These novel mutations were predicted to be deleterious by Polyphen-2 analysis as these residues are highly preserved through evolution. The phenotype associated with these mutations is indistinguishable from the previously reported mutations discussed above. 

TACR3 and TAC3 gene mutations have been first described by us (5). In a large multiethnic study, Gianetti et al (30) found TACR3 mutations in 5.5% of the whole cohort consisting of 345 nIHH probands. Among them, four out of 37 Turkish probands had TACR3 mutations (10.8%). However, it is not clear how many of these Turkish probands came from multiplex families. In 2011, Francou et al (31) identified three families with nine patients carrying TAC3 or TACR3 variants (5.2%) among 173 nIHH cases. In that article, there was only one multiplex family who showed a homozygous TACR3 deletion (c.483_499del). In our study, we have identified mutations in the TACR3 gene in six of the 22 families (27.2%), a frequency, significantly greater than the previously reported rates for the TACR3 gene mutations. One possible explanation for this could be that our probands come from clearly autosomal recessive pedigrees.

In summary, a significantly higher percentage of patients with familial nIHH have monogenic mutations when compared to sporadic cases. GNRHR and TACR3 mutations are the most common two mutated genes to account for the nIHH. "Identification of mutations in consecutive patients in familial cases of nIHH in a set time period in a highly consanguineous society may provide true picture of genetic etiology as well as potential for novel genes taking part in pubertal process." 

## Figures and Tables

**Table 1:**
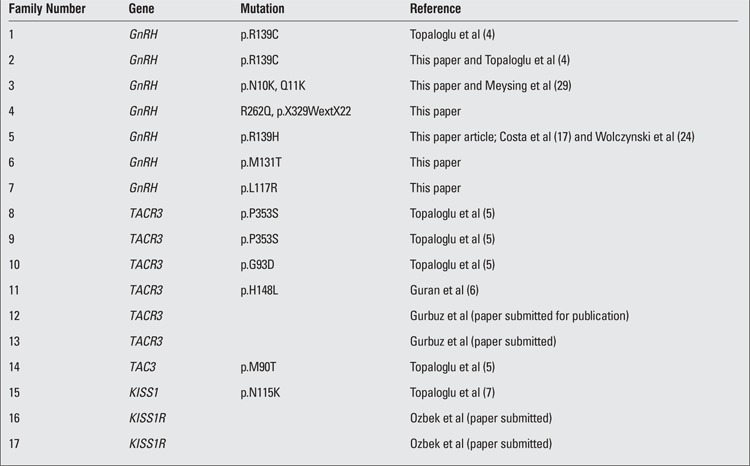
Reference data on families with gene mutations associated with nIHH phenotype
